# Isolated Celiac Trunk Dissection as a Cause of Abdominal Pain

**DOI:** 10.7759/cureus.72410

**Published:** 2024-10-26

**Authors:** Sergio L Jaramillo Escobar, Nicolás D Rosales Parra, René F Timarán Rodríguez, Daniela Giraldo Campillo, José D Puerta Rojas

**Affiliations:** 1 General Surgery, University of Antioquia, Medellín, COL; 2 Vascular Surgery, University of Antioquia, Medellín, COL; 3 Department of Internal Medicine-Vascular Specialist, University of Antioquia, Medellín, COL

**Keywords:** abdominal pain, blood vessel dissection, celiac artery, conservative management, splenic infarction

## Abstract

Isolated celiac trunk dissection is a rare condition involving dissection of this artery without aortic or other visceral artery involvement. We present an atypical case of a 46-year-old male smoker, hemodynamically stable upon admission, who experienced severe left hypochondrium pain. Imaging studies revealed narrowed flow in the celiac trunk and multiple splenic infarctions. After diagnosing isolated celiac trunk dissection, conservative management was successfully implemented. Surgical interventions are reserved for patients with hemodynamic instability, extensive ischemic involvement, or failure of conservative management.

## Introduction

Isolated celiac trunk dissection is a rare condition that predominantly affects men with a male-to-female ratio of 5:1 and an average age of 55 years. The etiologies involved include iatrogenic causes, atherosclerosis, trauma, smoking, cystic adventitial disease, pregnancy, fibromuscular dysplasia, inflammatory or infectious diseases, and congenital defects of the arterial wall. However, in many cases, the cause remains undefined [1,2]. In contrast to aortic dissection, which occurs between the two layers of the intima, consisting of the endothelium and the internal elastic layer, celiac trunk dissection occurs between the intima and the external elastic layer of the media [2].

## Case presentation

A 46-year-old male with a 30-pack-year smoking history presented to the emergency department with severe left hypochondrium pain that had persisted for one week with no other associated symptoms. Upon admission, he was hemodynamically stable. On physical examination, tenderness was noted in the left flank without signs of peritoneal irritation.

**Figure 1:**
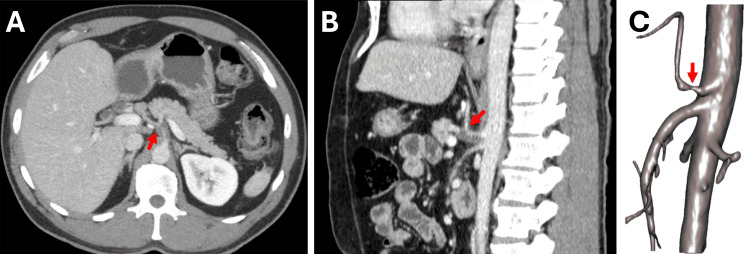
Contrast-Enhanced Computed Tomography Angiography A: Axial view, red arrow indicating the celiac trunk with a thrombosed false lumen and a patent true lumen. B: Sagittal view, red arrow indicating the celiac trunk with a thrombosed false lumen and a patent true lumen. C: Three-dimensional reconstruction, red arrow indicating narrowing of the true lumen.

A contrast-enhanced abdominal computed tomography scan revealed multiple splenic infarctions involving approximately 50% of the organ, along with narrowed flow at the origin of the celiac trunk (Figures 1A-1C). Tests to rule out antiphospholipid syndrome were conducted and returned normal, and additional tests for syphilis, HIV, and hepatotropic viruses were also negative.

Subsequent magnetic resonance imaging of the abdomen confirmed the presence of a dissection flap in the celiac trunk. The patient was treated with low molecular weight heparin and discharged after symptomatic improvement, with follow-up scheduled.

## Discussion

Isolated celiac trunk dissection is often asymptomatic and detected incidentally in imaging studies. Acute abdominal pain is the most common symptom, reported in 91% of symptomatic patients [3]. This pain is caused by compromised splenic, renal, or intestinal perfusion due to the extension of the dissection into other vascular beds. In chronic cases, symptoms like weight loss and postprandial pain are more frequent [2].

**Figure 2:**
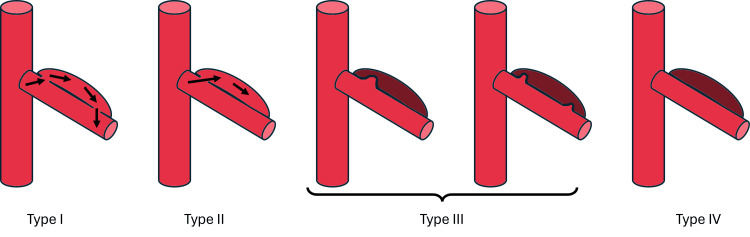
Classification of Spontaneous Dissection of the Superior Mesenteric Artery Type I: Patent false lumen with both entry and re-entry; Type II: False lumen shaped like a "cul-de-sac" without re-entry; Type III: Thrombosed false lumen with an ulcer-like projection (ULP), defined as a blood-filled pouch protruding from the true lumen into the thrombosed false lumen; Type IV: Completely thrombosed false lumen without ULP. Image created by the author.

Contrast-enhanced computed tomography angiography is the primary diagnostic tool, revealing findings such as a dissection flap or eccentric mural thrombus, which may be the only visible sign in some cases [2]. In acute presentations, perivascular fat stranding can also be observed. While classifications based on computed tomography findings [4], especially for superior mesenteric artery involvement (Figure 2), exist, these do not determine treatment but are useful for characterization. The most common presentation is a thrombosed false lumen without an associated ulcer.

There is no current consensus on the treatment of this condition. In the absence of perfusion compromise or active bleeding, conservative management is preferred. Medical management may include antiplatelet therapy, anticoagulation, or a combination of both [1]. Recommendations vary by symptoms, with antiplatelet therapy suggested for asymptomatic patients and anticoagulation for symptomatic individuals [3]. Blood pressure control is also crucial in conservative management. There are reports of retroperitoneal bleeding in hemodynamically stable patients who have benefited from conservative management with favorable outcomes [5]​​​​​​​.

Studies on isolated superior mesenteric artery dissection have shown no superiority of antithrombotic or anticoagulation therapy compared to observation, and some authors have extrapolated this evidence to the celiac trunk [6,7]​​​​​​​. In cases of acute complications or mesenteric angina, surgical or endovascular intervention may be required. Surgical management involves resection of the affected segment with primary or graft reconstruction, while endovascular treatment involves the use of covered and self-expanding stents [1]​​​​​​​. Stents with diameters of 6-8 mm and lengths of 40-60 mm are recommended [8]​​​​​​​, along with dual antiplatelet therapy for 3-6 months.

Follow-up with computed tomography angiography at six months and annually thereafter is suggested until dissection resolution. This is important considering that associated aneurysms occur in up to 50% of cases, with an elevated risk of rupture when the diameter reaches or exceeds 20 mm [1].

## Conclusions

Isolated celiac trunk dissection is a rare cause of abdominal pain, often presenting with well-defined findings on contrast-enhanced tomography, such as a dissection flap or eccentric mural thrombus. In most cases, conservative management is sufficient for stable patients, though surgical or endovascular intervention may be necessary for complications. This case underscores the importance of early diagnosis, appropriate imaging, and individualized treatment, as well as the need for long-term follow-up due to the potential for aneurysm formation and rupture.
